# Clinical Application of the Socket-Shield Concept in Multiple Anterior Teeth

**DOI:** 10.1155/2018/9014372

**Published:** 2018-06-20

**Authors:** G. Esteve-Pardo, L. Esteve-Colomina

**Affiliations:** Private Practice at Clínica Dental Esteve SL, Group Aula Dental Avanzada, Alicante, Spain

## Abstract

A case of rehabilitation of the upper front teeth is presented. To prevent bone resorption following extractions, a socket-shield technique on all the extracted teeth was performed. The combination of a staged extraction approach, the sequence of provisionals together with the minimal bone loss of vestibular volume, allowed solving this high aesthetic demanding case in a satisfactory way for the patient both in duration of the treatment and in its final outcome.

## 1. Introduction

**Figure 1:**
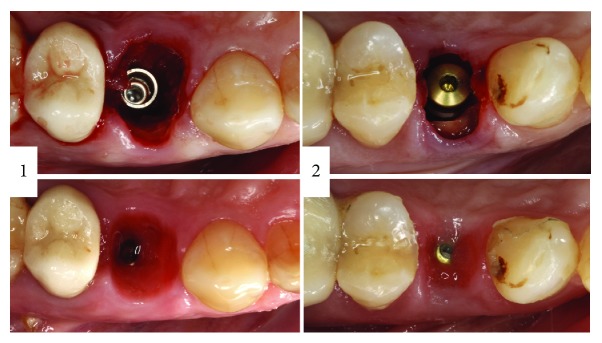
Two cases treated by immediate implant with SST and their occlusal view after three months of healing.

**Figure 2:**
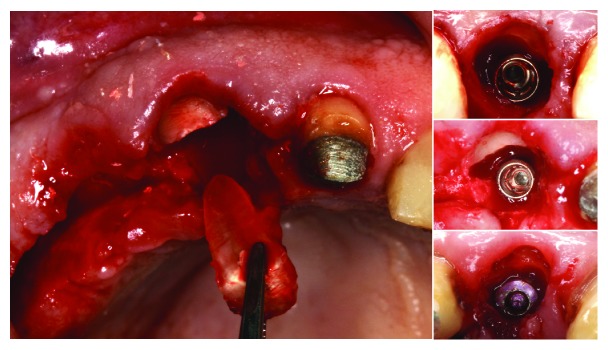
Different applications of the SST.

The socket-shield technique (SST) was first described by Hürzeler et al. [1]. The procedure consists of leaving a root fragment when extracting the tooth, specifically the vestibular portion of the most coronal third of the root (Figures 1 and 2).

It is widely known that following the extraction of a tooth a dimensional modification of the ridge is going to happen. This unavoidable and irreversible shrinkage is very unfavorable from the restorative point of view, especially in the aesthetic area. After three months, horizontal and vertical contractions of the alveolar volume occur [2] and these changes affect both to the soft and hard tissues [3].

The SST is aimed at making up for this loss of the vestibular volume “misleading” the bundle bone since the periodontal ligament remains attached to the dentine and cement of the root fragment.

Various animal studies demonstrated that the postextraction loss of volume could be highly diminished when leaving a tooth fragment attached to the cortical bone in the vestibular part of the alveolus [1, 4, 5].

The SST is yet missing clinical long-term data to be recommended as a standard treatment. A recent systematic review showed that the documentation on SST is reduced to some short-term case reports and case series and only a case-control study [6]. For the moment, the clinician has only his or her individual expertise as a criterion to decide when and how to apply this technique. From 2010, several variations of the original technique have been proposed [7]. The SST is beginning to be considered as one type of partial extraction therapies (PET) [8], a concept derived from the root submergence technique (RST) initially proposed by Salama and coworkers for pontic site development [9].

The partial extraction of a tooth is a complex procedure since the tooth fragment to leave should not be luxated at all by the movements used to extract the rest of the root [10]. Otherwise, the following complications may occur: loss of the tooth fragment, resorption of vestibular bone, infection, exposure of implant threads, and even implant failure. All these could worsen the situation of having extracted the whole tooth completely [6].

The traditional way to try to compensate the loss of vestibular volume in an immediate postextraction implant has been hard and soft tissue grafting [3]. We should not see the SST as a substitute for it, but rather as a complement when it can be carried out. It seems to have advantages compared to the connective tissue graft (CTG), but this issue is beyond the scope of the article.

This case report will show a clinical case where immediate implant placement in the aesthetic area was performed using the SST. The sockets not to be implanted and receiving the pontics were treated by alveolar preservation with the SST. This way a successful aesthetic restoration was achieved as the tissue volume seems to be maintained.

## 2. Case Description

**Figure 3:**
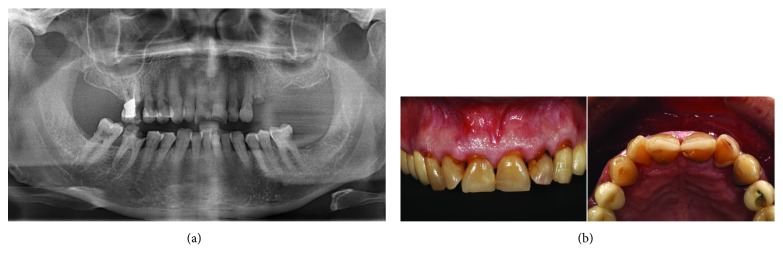
(a) Initial panoramic radiograph. (b) Clinical view of the anterior teeth. The roots are subgingivally and peripherally decayed.

The patient was a 76-year-old man who came to the office in 2014 looking for possible treatments of his fractured central incisors. Nothing was found relevant about his medical condition. The patient shows a high risk for caries and also eccentric bruxism. He has partial edentulism in the superior left quadrant and multiple decay and fractured teeth. The initial approach was conservative aiming to keep the upper front by means of composite fillings (Figures 3(a) and 3(b)). Then, the posterior superior quadrants needed to be restored with implants.

**Figure 4:**
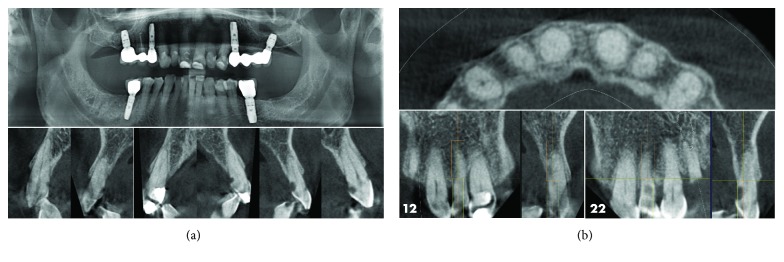
(a) Panoramic X-ray. (b) 3D slices showing the implant planning. The root caries can be seen.

Three years after, in 2017, the patient came back to the office referring pain of endodontic origin in the upper left canine. New and secondary subgingival caries were found in the six front teeth. The conservative prognosis was considered poor due to the subgingival depth and extent of decay presented by the lesions from canine to canine. After having discussed the treatment options, especially the surgical lengthening of the front teeth or the orthodontic extrusion, the patient decides to replace the residual teeth with a new implant-supported bridge similar to the recently performed prostheses of the posterior areas that were judged by him as a highly satisfactory treatment. The patient preferred not to involve these restorations in the present anterior treatment and limited it to place only two implants in the lateral incisors' positions (Figure 4).

The treatment was carried out in a staged approach. Briefly, first, we extracted the lateral incisors, using the SST, and placed two immediate implants. The four residual teeth were then prepared to be used as abutments of a temporary bridge for the purpose of maintaining the aesthetics and function of the patient during the early osseointegration period. In a further step, the four remaining teeth were also extracted using the SST, and the initial provisional bridge was replaced by the second provisional screwed on the uncovered implants. Only one out of the four abutment teeth used for the temporization of root canal treatment was needed due to a periapical infection.

**Figure 5:**
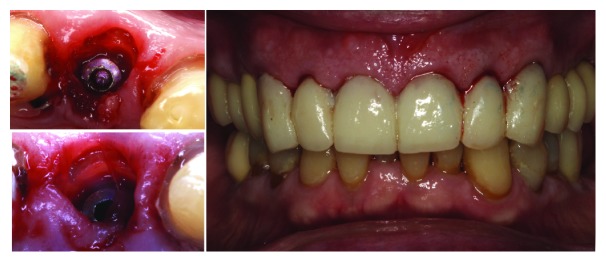
Stage one: implants placed with SST in the lateral incisors' sites and the immediate temporary bridge on the abutment teeth.

When placing the two immediate implants into the alveolus of the lateral incisors, a section of the buccal part of the root (about the two middle thirds) was left in place and no biomaterial was used at all. An impression of the implants was taken to have the second temporary bridge available in the second surgery. Healing abutments were then attached with the proper height for the soft tissue to cover them but at the same time facilitating the uncovering. Finally, a temporary acrylic bridge was cemented onto the four abutment teeth 13-11 and 21-23 (Figure 5).

**Figure 6:**
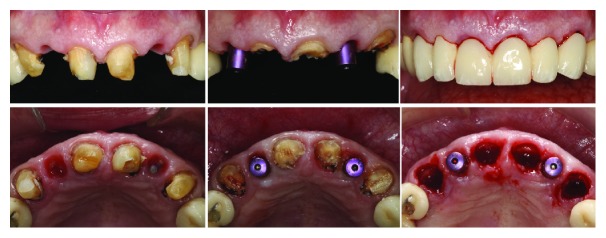
Stage two: partial extraction of the remaining teeth and placement of the second provisional onto the uncovered implants.

Three months later, the implants were uncovered, the four abutment teeth were extracted, again with the SST—partial extraction of the roots—but this time no more implants were placed in these sockets. The first provisional cemented onto the teeth was then replaced by a second acrylic bridge screwed onto the implants though temporary abutments (Figure 6).

The partial extraction of the canines, aiming to leave a buccal slice of the root, was so hard to perform, and further instrumentation would lead to the socket destruction that a decision was intraoperatively made and a greater portion of the root, including the apex, was finally left. As the locations of the canines did not involve the implant sites, any potential complication could be addressed efficiently.

**Figure 7:**
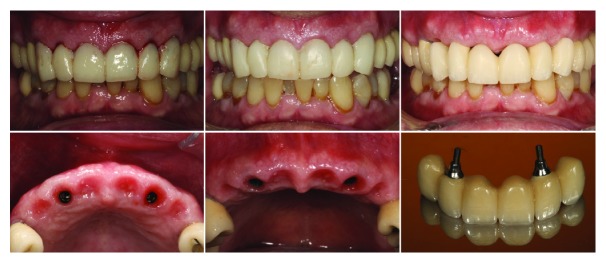
Frontal view of immediate provisional after implant placement, 3 months after healing and 1 month after 2nd implant provisional prosthesis. Soft tissue view before prosthodontic phase.

**Figure 8:**
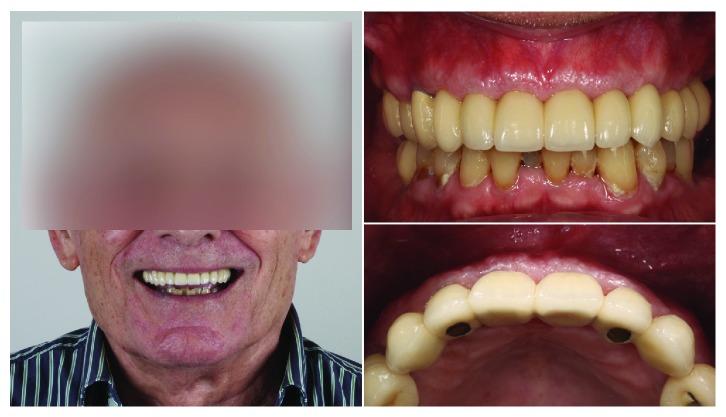
Final restoration in place and its integration on the patient smile.

**Figure 9:**
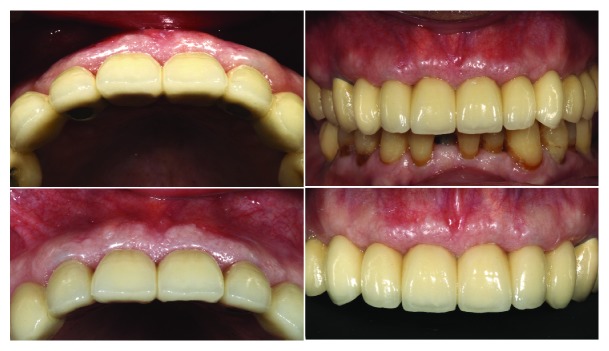
Aesthetic appearance after 6-month follow-up.

One month later, the prosthodontic phase was undertaken. Little if any differences in the buccal tissue volume and no noticeable aesthetic impact could be found after the multiple extractions (Figure 7). The desired position of the incisal border was determined by various try-ins, and five months after implant surgery, the definitive prosthesis was placed. The final clinical aspect can be appreciated in the pictures (Figures 8 and 9).

## 3. Discussion

There is still insufficient evidence to support the SST with simultaneous implantation. Only a few case reports are available showing variable data of bone loss. In a case-control study in 2014, a medium vertical bone loss of 0.8 mm was reported in 26 implants on 25 patients after 24 months of follow-up [11]. In a prospective clinical case series study, the marginal bone loss was reported to be 0.7 mm on average after 6 months [12]. In a retrospective study on 10 patients in 2017, a mean bone loss of 0.33 mm in mesial and 0.17 mm in distal were reported [7].

In a recent systematic review, the authors find a horizontal bone loss of 1.07 mm and 0.78 mm vertically after the immediate placement of implants [13]. Usually this horizontal bone loss has to be compensated by bone augmentation and/or a connective tissue graft [14].

Although the amount of marginal bone loss in the SST is still not conclusively proved, current clinical experiences seem to point to a minimal, negligible, or even not existent bone loss after extraction. As a consequence of this, soft tissue grafting would not be necessary in most of the patients treated by this technique. In the aforementioned case-control study in 2014, the authors found a significant difference in aesthetic impact when comparing the socket shield to the conventional technique [11].

Needless to say that if grafting is not an aesthetic requirement to compensate the horizontal bone loss, the treatment becomes more patient-friendly with less duration and morbidity. Nevertheless, the SST is an operator-sensitive procedure, delicate to handle, and sometimes very hard to perform [15].

In this case, the first provisional bridge on abutment teeth allowed the patient to comfortably wear a fixed temporary prosthesis during the healing time of the immediately implanted sockets. This bridge was not used to shape the soft tissues. The staged extraction approach avoided a major tissue loss and contributed to maintain a more aesthetic tissue architecture [16].

To support, a 6-unit prosthesis by only two implants and with two cantilevers in the canine positions could also be a reason for discussion. Another option previously discussed with the patient was a full-arch prosthesis splinting the two new implants to the four preexisting ones. The patient was satisfied with the recently restored posterior quadrants and rejected it. A three-fixed superior rehabilitation scheme allowed us to perform a simpler treatment with better acceptance by the patient. Given the evident bruxism, the number of implants could be considered low for the anterior bridge—six teeth on two implants—but there is a growing clinical evidence about lower number of implants to support a full arch. Should a proper occlusion is achieved and the patient wears an occlusal splint, the distal cantilevers seem not to be a problem [17].

**Figure 10:**
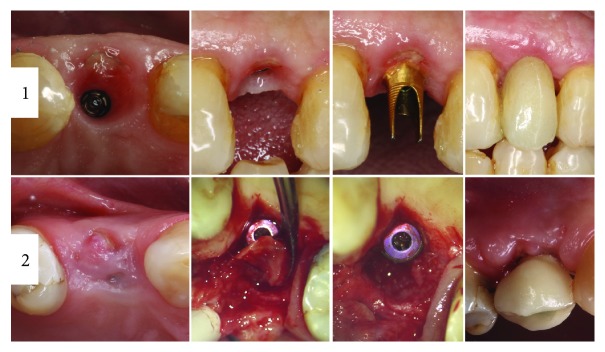
Two cases with SST complications. In 1, lateral incisor restoration with the shield communicated with oral cavity. In 2, first premolar with luxated shield on implant second stage. Implant failed at the last case after 4 months.

Since decades, clinicians have been trying to avoid the loss of alveolar volume by leaving root remnants [18]. In an old study on 2000 patients, the authors reported that a 16.2% of the root remnants resulted in pathological condition signs especially when exposed to the oral environment [19]. Although numerous papers since the late seventies dealt with the so-called “root submergence technique,” this still remains a controversial issue. The uneventful healing of sockets with root fragments has been well documented [20]. Both vital tooth retention [21, 22] and submergence of endodontically treated roots [23, 24] have been recommended to prevent excessive resorption of the residual ridge. This concept has been recently applied to teeth- or implant-supported fixed prostheses for pontic site development [9, 25–27]. Based on this background, a decision was made to leave the canine roots instead of performing a more invasive surgical procedure for extracting them. One of the main factors for the success of the SST is precisely that the root fragment does not come in contact with the external medium [7], something that could facilitate the infection and also be an aesthetic problem (Figure 10).

A human histologic study has been recently published demonstrating osseointegration between an implant surface and a dentin surface of a root fragment from a SST making the technique further promising [28].

## 4. Conclusion

The SST has currently not enough clinical evidence for being recommended as a routine option. It seems that if the proper clinical requirements are met and the technical handling of the operator is appropriate, the SST could minimize the resorption of the buccal tissues after the tooth extraction. In selected cases, the immediate placement of implants with the SST seems to be a useful tool for the replacement of the teeth lost, especially in the aesthetic area.
